# Assembling Ordered Nanorod Superstructures and Their Application as Microcavity Lasers

**DOI:** 10.1038/srep43884

**Published:** 2017-03-08

**Authors:** Pai Liu, Shalini Singh, Yina Guo, Jian-Jun Wang, Hongxing Xu, Christophe Silien, Ning Liu, Kevin M. Ryan

**Affiliations:** 1 Department of Chemical Sciences and Bernal Institute, University of Limerick, Limerick, Ireland; 2 Bernal Institute University of Limerick, Limerick, Ireland; 3 The School of Physics and Technology, The Institute for Advanced Studies and The Center for Nanoscience and Nanotechnology, Wuhan University, Wuhan, 430072, China; 4 Department of Physics and Bernal Institute, University of Limerick, Limerick, Ireland

## Abstract

Herein we report the formation of multi-layered arrays of vertically aligned and close packed semiconductor nanorods in perfect registry at a substrate using electric field assisted assembly. The collective properties of these CdSe_x_S_1-x_ nanorod emitters are harnessed by demonstrating a relatively low amplified spontaneous emission (ASE) threshold and a high net optical gain at medium pump intensity. The importance of order in the system is highlighted where a lower ASE threshold is observed compared to disordered samples.

Colloidal nanocrystal lasers offer significant promise for low cost, high gain devices that can be synthesized and processed from solution. 1D rod-shaped nanocrystals (rods, wires, belts) are of particular interest as they have shown a lower threshold for lasing in addition to exhibiting directional and polarized emission^1^,^2^. The knowledge gained from quantum dot solids indicates that close packing is necessary to allow sufficient volume fraction of nanocrystals to overcome losses due to non-radiative processes and allow ASE^3^,^4^,^5^. Therefore harnessing the collective emission from colloidal semiconductor nanorods through organized assembly has enormous potential for next generation lasers with particular interest for their application in integratable nanophotonics as components for all-optical integrated circuits^6^,^7^. Previously, optically pumped lasing has been observed where order occurs in dot-in-rod ensembles, cylindrical microcavities, and self-assembled ring patterned arrays^8^,^9^. Fully harnessing the enhancement from assembly requires complete orientational and positional order of the nanorod emitters across an entire substrate to allow for a practical and reproducible route to optical integration.

Significant progress has been made in the hierarchical organization of semiconductor nanorods into 1D, 2D, and 3D arrangements^10^,^11^,^12^ using both self and directed protocols, where in all cases the particle-particle interactions affected by the charge and dipole moment of the rod plays a role^13^,^14^,^15^,^16^. We have shown that aligned assemblies with a high degree of order can be effectively grown from a substrate using electric field assisted assembly^17^,^18^. The net nanorod charge confers electrophoretic mobility on the rods under the influence of the applied field whereas the intrinsic dipole moment ensures orientation of the rods along the field lines. Previously, generating strong PL emission in the rod shape was possible by forming the CdSe-CdS dot-in-rod structure developed by Manna *et al*.^19^. More recently, we have achieved strong PL emission in the cadmium chalcogenides system without a core-shell structure by combining the metal cation with both chalcogen anions in an alloy^20^. This alloyed nanostructure allows precise tuning of the bandgap as a function of anion ratios allowing for optimization as needed for light emitting applications^21^,^22^.

Here, we form thickness and compositionally controlled assemblies of CdSe_x_S_1-x_ alloyed nanorods directly at substrates using electric field assembly. The influence of order results in a 36% lower ASE threshold and a high net optical gain, determined using variable stripe measurements, in these wavelength tunable assembled films. For CdSe_0.625_S_0.375_ nanorods, a net gain of 24 cm^−1^ is obtained on the highly ordered sample at pump intensity of 35.5 mJ/cm^2^. Moreover, a net gain of 59 cm^−1^ is achieved on the CdSe ordered film at 44.1 mJ/cm^2^. In particular, lasing from a square cavity (10 μm × 10 μm- patterned by focused ion beam milling) in the assembled film is demonstrated. Their ease of assembly from solution, controllable deposition procedures and subsequent compatibility for further processing makes this route an attractive approach for integratable nanophotonics.

## Results

**Figure 1:**
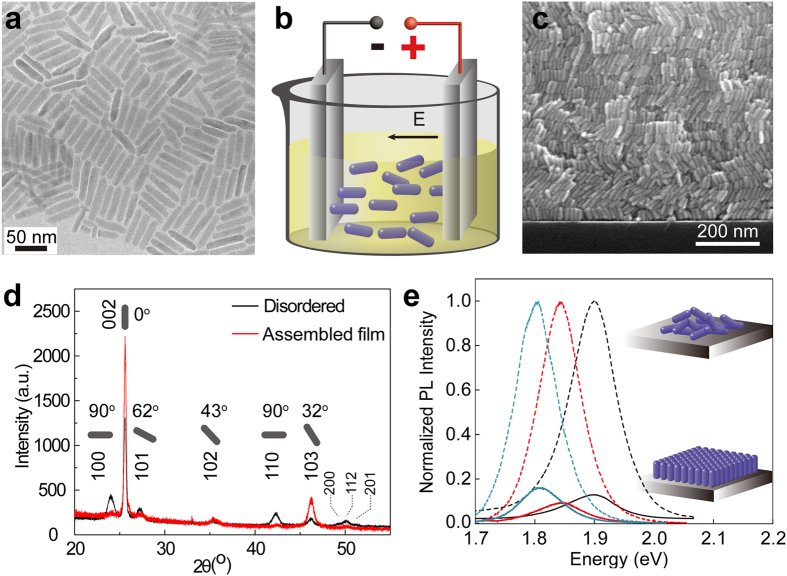
Experiment setup and morphology characterizations of CdSeS nanorods film. (**a**) TEM image of CdSe_0.625_S_0.375_ nanorods with an aspect ratio of 4; (**b**) The setup for electric field assembly; (**c**) Cross-section SEM image of vertically aligned multilayer nanorods on a Si substrate. (**d**) XRD patterns the aligned film (red line) and disordered nanorods (black line); (**e**) PL characterization of assembled (solid line) and disordered (dash line) films in three compositions: CdSe (blue line), CdSe_0.625_S_0.375_ (Se-rich sample, red line), and CdSe_0.375_S_0.625_ (S-rich sample, black line).

The CdSe_x_S_1-x_ nanorods capped mainly by alkylphosphonic acid were synthesized via a modified method^20^ where *x* could be tuned from 1 to 0.375 while maintaining a highly monodisperse distribution of rods with average aspect ratio γ = 4 (Fig. 1(a)). The nanorods were dispersed in toluene and deposited under a DC field (Fig. 1(b)), forming a film on the anode that grows according to the deposition time (0.1~2 μm thick). The high magnification SEM image shows that the nanorods are packed in a highly ordered manner with each nanorod *c*-axis orthogonal to the substrate having aligned with the applied field direction (Fig. 1(c), SEM of an aligned CdSe nanorods sample is shown in Figure S1(b) and Figure S6(b)). The XRD pattern of the aligned film (red line in Fig. 1(d)) shows a substantial increase in the intensity of the (002) peak with regards to the disordered sample, with a concomitant reduction in the (100), (101) and (110) reflections. This occurs because the nanorods whose long axis grows along the <002> direction are standing vertically on the substrate, where only the (002) plane and its parallel planes diffract^16^,^23^. Rietveld Refinement of the XRD pattern is shown in Figure S6(a), with 83.7 ± 6% of the nanorods in the sample are parallel to the <002> direction.

To demonstrate the bandgap tunability of assembled films, the solid state PL of three samples of different compositions in both disordered and ordered states are shown in Fig. 1(e). The band gap shifts from 1.8 eV to 1.9 eV according to Se/S composition at *x* = 1, 0.625 and 0.375, respectively. A quench (80~90%) of PL intensity and a five-fold reduction in fluorescence lifetime are observed on transitioning from disordered samples made by drop-casting to highly ordered samples using the same batch of rods (Figure S2). A similar phenomenon has been observed previously in dyes where rhodamine 6G at high concentration undergoes a self-quenching effect, due to the formation of dimers or other quenching complexes, which have nonradiative deexcitation channels^24^,^25^. As nanorods are aligned parallel to each other with a rod-to-rod distance of 1~2 nm, both radiative and nonradiative decay rates are likely to be modified considerably due to the induced radiative scattering and absorption in their neighbors, which can lead to considerable changes in PL intensity and lifetime^26^. Additionally, the backscattered configuration used in our experiment mitigates against the collection of light from the directional emission of vertical alignment of the nanorods, which could also contribute to a reduction in collected PL^27^.

**Figure 2:**
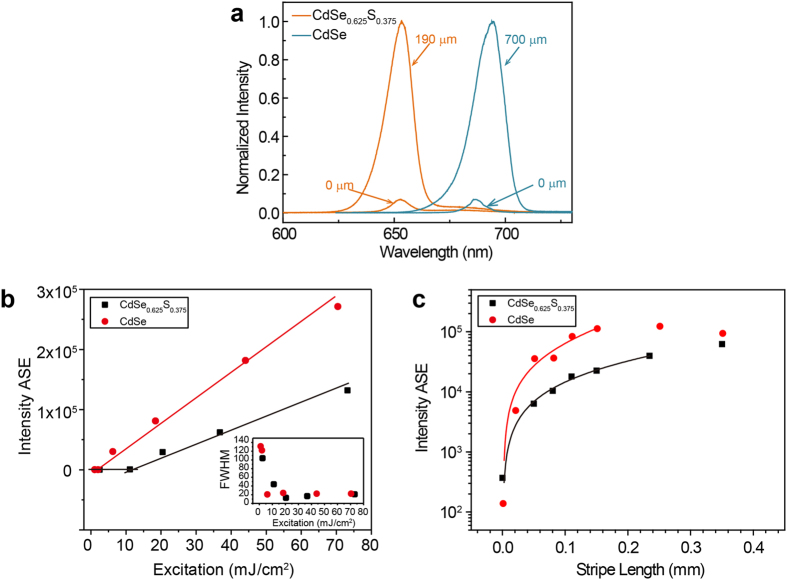
Photoluminescence characterization. (**a**) ASE spectra obtained from CdSe_0.625_S_0.375_ (Se-rich sample, orange line) excited over lengths between 0 and 190 um at 22.7 mJ/cm^2^ and CdSe (blue line) excited over lengths between 0 and 00 μm at 3.37 mJ/cm^2^; (**b**) ASE intensities for CdSe_0.625_S_0.375_ and CdSe nanorods at stripe length of 0 mm and 1.05 mm; the inset is the corresponding FWHM of data in (**b**); (**c**) ASE intensities as a function of stripe length for CdSe_0.625_S_0.375_ and CdSe nanorods at pump intensity 35.5 mJ/cm^2^ and 44.1 mJ/cm^2^ respectively.

Nevertheless, the quenching in fluorescence lifetime does not have a significant impact on the assembled nanorods as a highly suitable material for optical gain applications. To obtain the ASE threshold, a cylindrical lens is used to focus a pulsed laser beam at 563 nm (1 KHz, ~5 ps pulse width) into a stripe of 2 mm × 10 μm on the sample with thickness ~600 nm (Figure S3). The PL spectra of the ordered samples collected from the end of the stripe are recorded as a function of the pump energy. The ASE spectra obtained at difference stripe lengths of two samples are given in Fig. 2(a) for comparison. The transition from spontaneous emission to ASE can be clearly identified as the PL intensity demonstrates an abrupt increase accompanied by the rapid narrowing of the full width at half maximum (FWHM) of the PL band with the increase of pump energy (Fig. 2(b)). The ASE thresholds are obtained where linear fits to the spontaneous emission and ASE intersect each other, and the values are 2.05 mJ/cm^2^ and 11.14 mJ/cm^2^ for CdSe and CdSe_0.625_S_0.375_ nanorods assembled films, respectively. PL of the ordered sample comprised of CdSeS (*x* = 0.375) is also shown in Figure S4, where the ASE threshold is 6.91 mJ/cm^2^. To estimate the net optical gain of these materials at various excitation energies, the ASE intensity (*I*_*AS*E_) on the ordered samples is recorded as a function of stripe length *L*^28^,^29^.


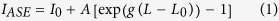


The net optical gain coefficient *g* is obtained by fitting the data in Fig. 2(c) to eq. 1, where *I*_*0*_, *A* and *L*_*0*_ are fitting parameters. Notably, the net gain becomes saturated for the stripe length longer than 0.23 mm for the CdSeS and 0.15 mm for the CdSe nanorods. Right above the ASE threshold, the gain coefficient for CdSe_0.625_S_0.375_ nanorods is relatively low, around 1.35 cm^−1^ at a pump energy of 22.7 mJ/cm^2^. At pump energy of 35.5 mJ/cm^2^, however, a net gain of 24 cm^−1^ is obtained on the CdSe_0.625_S_0.375_ ordered sample, and a net gain of 59 cm^−1^ is achieved on the CdSe ordered film at 44.1 mJ/cm^2^, as shown in Fig. 2(c). For the disordered samples, 36% higher pump energy is required for the sample to reach ASE (The PL revolution of both ordered and disordered sample can be found in Figure S5). At the threshold of ASE, the internal optical gain is expected to reach the same level as the total loss. We therefore attribute the higher energy requirement to reach ASE for the disordered samples to the lower volume packing density and higher scattering loss due to the disordered nature of the sample (Figure S6)^30^.

**Figure 3:**
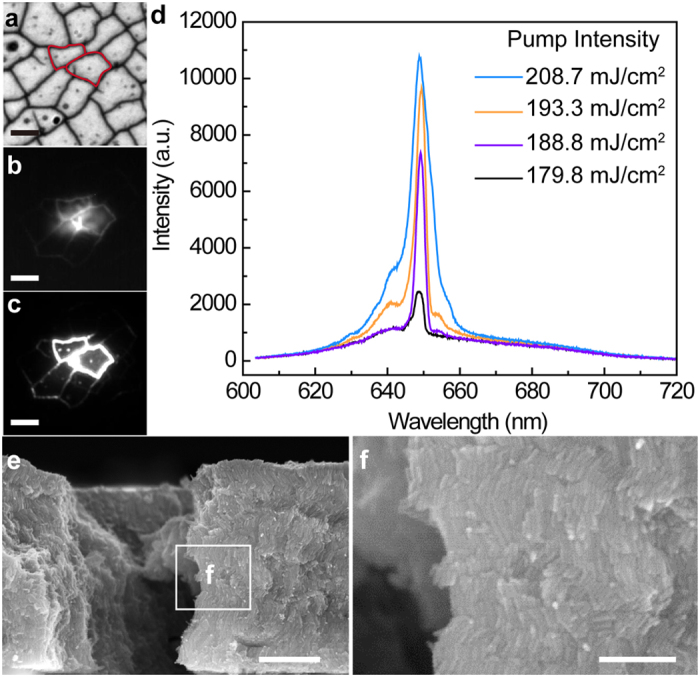
Lasing from a cracked CdSe_x_S_1-x_ film. (**a**) Wide field optical image recorded in the reflective mode of cracked film; (**b**,**c**) Images recorded on the same area with pump power at 138.4 and 560.6 mJ/cm^2^ respectively, after 600 nm long pass filter (scale bar is 20 μm): (**d**) Evolution of PL of a cracked CdSe_0.625_S_0.375_ film with various pump power; (**e**) and (**f**) are cross-section SEM images at different magnification of cracks in a 700 nm thick film (scale bar of (**e**) is 500 nm, and scale bar of (**f**) is 200 nm).

**Figure 4:**
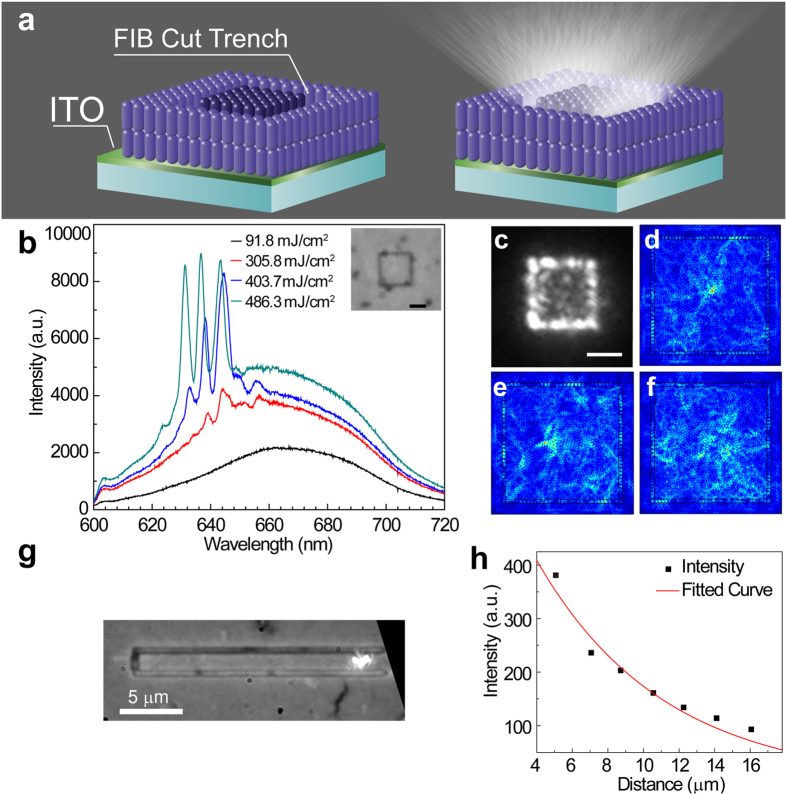
The patterned microlaser. (**a**) Schematic of cut trench on assembled film and lasing; (**b**) The evolution of PL spectra of the square cavity fabricated cut in anordered CdSe_0.625_S_0.375_ film and the onset of lasing. The inset shows a wide field optical image of the square. (Scale bar is 5 μm); (**c**) The image recorded in the same area with pump intensity at 486.3 mJ/cm^2^ (Scale bar is 5 μm); (**d**–**f**) Simulated mode distribution at wavelengths 641.6 nm, 634.6 nm, and 629.3 nm, respectively; (**g**) Optical microscopy image of FIB patterned waveguide and (**h**) the PL emission observation of the propagation loss of the film.

When the thickness of the sample is over ~800 nm, some of the assembled films start to crack due to strain within the film during solvent dewetting (Fig. 3(a))^31^. These features act as natural feedbacks to the propagation of light, and random lasing can be observed from the cracks. Figure 3(b and c) are images recorded on the same area with pump intensity at 138.4 and 560.6 mJ/cm^2^ after a 600 nm long pass filter, corresponding to spontaneous emission and lasing. The evolution of PL as a function of pump intensity giving rise to the lasing peak is shown in Fig. 3(d). The cross-section SEM images in Fig. 3(e and f) show that the alignment of rods is not disturbed by the occurrence of cracking. To further demonstrate the capability of the ordered nanorod film as a suitable lasing material for integrated photonics applications, we patterned a square shaped microcavity (10 µm × 10 µm). This was achieved on a 600 nm thick, ordered CdSe_0.625_S_0.375_ nanorod film on an ITO/Glass substrate using a FIB to mill out a 300 nm wide trench around the square (as outlined in the schematic Fig. 4(a)). The edges of the square provide the feedback in the form of a resonant cavity. Figure 4(b) shows the evolution of PL emission as a function of pump intensity on the patterned square, which clearly demonstrates the emergence of resonant cavity modes and the onset of lasing. The FWHM of the cavity modes is around 2 nm at a pump intensity of 403.7 mJ/cm^2^, indicative of lasing behavior. Figure 4(c) is the optical image of the lasing cavity recorded by a CCD camera with pump intensity at 486.3 mJ/cm^2^ after a 600 nm long pass filter where the scattered light is clearly observed from the cavity edges. Using theories developed by Poon *et al*.^32^, we estimate there are over 500 resonant modes within the emission band of CdSe_0.625_S_0.375_ nanorods (620 to 700 nm), in a 10 μm × 10 μm square cavity (See Supporting Information). Figure 4(d,e and f) give the simulated mode distribution in the cavity using COMSOL simulation package at three resonant wavelengths 641.6 nm, 634.6 nm, and 629.3 nm, respectively, as shown in Fig. 4(b). It is notable that multiple cavity modes are excited at each wavelength and the modes are mostly confined within the square. The lasing threshold of the milled structure is higher than that of the random lasing case, which we attributed to the damage to the materials during FIB patterning, which usually leads to a significantly higher overall loss.

To estimate the total propagation loss of the patterned structure, a waveguide of 2 μm in width and 20 μm in length was also patterned on the same sample using FIB (Fig. 4(g)). The plot in Fig. 4(h) was obtained by monitoring the PL emission (*I*) at the end of the waveguide as the distance (*R*) of the excitation-collection changes; the data are fitted with exponential decay curve I = 725.19exp(−R/7.138), yielding a loss coefficient of 1415 ± 143 cm^−1^. As the onset of lasing indicates that the internal gain exceeds the total loss, we expect that the internal gain of the assembled nanorods film is greater than this value. This indicates the potential of this material as a promising candidate in future high gain applications.

## Discussion

In summary, we have demonstrated lasing from highly ordered cadmium chalcogenide nanorod assemblies deposited at substrates using electric field assisted assembly. The architectural arrangement allowed the formation of a thickness controlled deposit comprised of layers of vertically aligned and close-packed nanorods, all oriented orthogonal to the substrate. The high degree of alignment of the nanorods in the ordered assembly manifested as a reduction of 36% in ASE threshold compared to that of a disordered sample. This provided sufficiently large optical gain to achieve lasing in the form of cavity modes in both natural crack propagations or in customized square cavities fabricated in the assembly. The assembled nanorod films provide a low cost way to achieve active optical functionalities and opens pathways to achieve integrated optics and optoelectronics that are can be easily fabricated by scalable wet-processing techniques.

## Methods

### Synthesis of CdSe_x_S_1-x_ nanorods

Se-S stock solution was prepared in advance: 0.0494 g of selenium (0.625 mmol, 99.99% Sigma-Aldrich) and 0.012 g of sulfur (0.375 mmol, >99.5%, Sigma-Aldrich) are dissolved in 500 μL trioctylphosphine (TOP, 97%, Sigma-Aldrich) forming a colorless solution. The basic synthesis route followed the as reported hot-injection method^20^. The mixture of 0.11 g cadmium oxide (>99%), 1.45 g of trioctylphosphine oxide (99%, Sigma-Aldrich), 0.11 g of hexylphosphonic acid (HPA, PCI Synthesis) and 0.44 g of n-octadecylphosphonic acid (ODPA, PCI Synthesis) were added into a three-neck round-bottom flask and heated to 120 °C, followed by evacuation under vacuum for 45 min. The temperature was then raised to 330 °C in an argon atmosphere where the color of the mixture changed to colorless. During heating, 1 ml of TOP was injected into the flask at 300 °C. Then 500 μL of the Se-S stock solution was rapidly injected once the temperature reached 330 °C, causing a gradual color change from colorless to reddish-brown. After injection, the growth continued for 15 min with stirring. Subsequently, the heating mantle was removed and the reaction vessel was allowed to cool down to 80 °C. The product was then transferred to a vial and washed with anhydrous toluene and isopropanol in 1:2 ratios and centrifuged in 4000 rpm for 10 min to remove excess ligands. The precipitate was collected and retained for deposition experiments.

### Electric field assisted assembly of CdSe_x_S_1-x_ nanorods

The as-washed nanorods were dispersed in 5 ml toluene as an electrolyte solution and sonicated for 3 min before deposition. The optimum ligand coverage was determined to be 2.912 wt.% from thermal gravity analysis results. Two pieces of Si substrates (30 mm × 10 mm, phosphorus doped, n-type, supplied by Silicon Materials) were attached on both sides of the spacer as electrodes. The electrodes were maintained at a distance of 2.15 mm in a parallel configuration to form a uniform electric field. To achieve the 500 nm and 800 nm thick samples, nanorods were deposited for 5 min under a DC field with a strength of 612 V/cm. The potentials were controlled and monitored by Technix High Voltage Supply and TTi 1604 Digital Multimeter. The electrodes were then raised and dried slowly in a toluene atmosphere with the assemblies forming on the negative electrode. For the optical characterizations, ITO substrates were used as the negative electrode.

### Characterizations

A Hitachi SU-70 scanning electronic microscope was used to confirm the alignment of the nanorods in films To characterize the optical gain of the CdSe_x_S_1-x_ nanorod film, a Coherent Libra (1 KHz, 100 fs) was used to pump a Light Conversion optical parametric amplifier TOPAS (1 KHz, ~4 ps). The laser output is tunable from 500 nm to 5 μm. In this experiment, the pump laser was focused on the sample using ×20 objective lens. The scattered and reflected light was collected by the same objective and was then redirected to a CCD camera or spectrograph.

The spontaneous emission lifetime of the CdSe, CdSe_x_S_1-x_ nanorod films were measured using a Time Correlated Single Photon Counting (TCSPC) method. To measure the lifetime of the PL, the sample was excited with a supercontinuum laser (fianium, 10 MHz, 76 ps pulse width) after passing through at 575 ± 20 nm band filter. The emitted photons from the sample were first filtered by a 630 long pass filter, and then directed into the single photon avalanche diode (SPAD). The induced electric pulses were then sent to the counting unit PicoHarp 300 for processing. After a certain accumulation time, the time trace of an optical pulse was reconstructed with a time resolution of 4 picoseconds.

### COMSOL Simulation

Wave optics module in COMSOL Multiphysics 4.4 simulation package was used to simulate the mode distribution at the chosen wavelength. Because of the large size of the square cavity, a 2D simulation was used instead of full field 3D simulation. Firstly, a 1D simulation was used to extract the effective mode index for the air-CdSe_x_S_1-x_-ITO cross section. The effective mode index in the 2D simulation was applied to obtain the mode distribution. Both the eigenfrequency and frequency domain studies were applied to get the mode distribution, which showed similar results at the chosen wavelengths. Due to the high number of eigenmodes within the cavity, there were multiple eigenfrequencies converged at a similar wavelength. The ones with mode distribution within the square cavity were plotted which have the highest quality factor in the converged solution, suggesting the smallest loss from the chosen mode.

## Additional Information

**How to cite this article:** Liu, P. *et al*. Assembling Ordered Nanorod Superstructures and Their Application as Microcavity Lasers. *Sci. Rep.* **7**, 43884; doi: 10.1038/srep43884 (2017).

**Publisher's note:** Springer Nature remains neutral with regard to jurisdictional claims in published maps and institutional affiliations.

## Supplementary Material

Supporting Information
