# Supplementary material for: Assembling Ordered Nanorod Superstructures and Their Application as Microcavity Lasers
Source: Sci Rep. 2017 Mar 8;7:43884. doi: 10.1038/srep43884 (PMC5341026; doi:10.1038/srep43884)
Supplement: Supporting Information [file srep43884-s1.pdf]

Supporting Information

Assembling Ordered Nanorod Superstructures and Their Application as Microcavity Lasers

Pai Liu[1], Shalini Singh[1], Yina Guo[2], Jian-Jun Wang[1], Hongxing Xu[3], Christophe Silien[4], Ning Liu[4]* and Kevin M Ryan[1]*

[1.] Department of Chemical and Environmental Sciences and Materials and Surface Science Institute, University of Limerick, Limerick, Ireland

[2.] Materials and Surface Science Institute (MSSI), University of Limerick, Limerick, Ireland

[3.] The School of Physics and Technology, The Institute for Advanced Studies and The Center for Nanoscience and Nanotechnology, Wuhan University, Wuhan, 430072, China

[4.] Department of Physics and Energy and Materials and Surface Science Institute, University of Limerick, Limerick, Ireland

* Corresponding authors: ning.liu@ul.ie and kevin.m.ryan@ul.ie

Control Experiment of Evaporation Assembly

A control experiment was carried out where the electrodes are dipped in solution without applying the electric field that would mimic a dip-casting process where solvent evaporation is used to consolidate particles to a substrate. The outcome of this experiment shows that, without electric field, there is hardly any deposition with only a few nanorods randomly deposited on the substrate. (Figure S1(a)) Therefore the electric field is the primary force for organizing of the rods at the substrate.

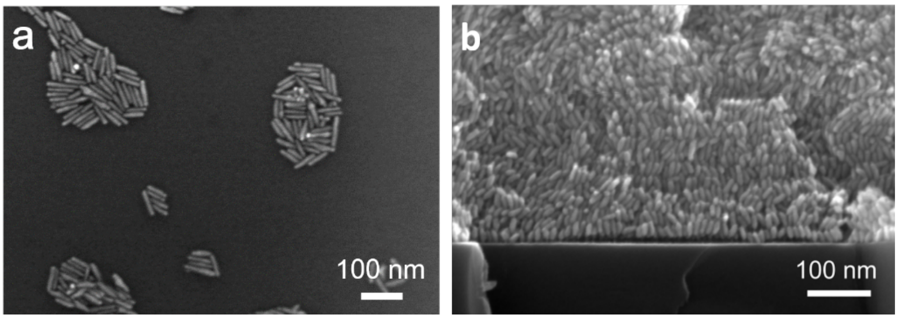

Figure S1. (a) SEM image shows that there is no continuous film deposited on the substrate when no field is applied ;(b)Cross-section SEM image of electric field aligned CdSe nanorods film.

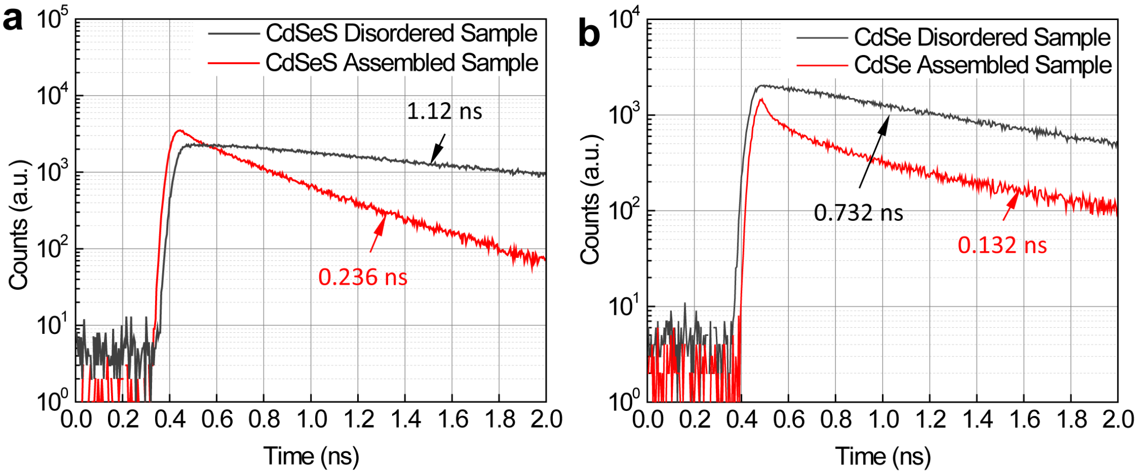

Figure S2. Photoluminescence decay of (a) CdSeS (Se/S=5:3) and (b) CdSe nanorods assembled film (red line) and disordered film (black line).

Variable stripe measurements

To carry out the variable stripe measurements, the excitation laser was focused to the sample by a cylindrical lens. At the focal point, the width of the striped shaped beam is around 2 um, as shown in Figure S3(b). The position of the sample with respect to the striped beam is varied by a micrometre and the output PL from the edge of the sample is recorded and shown in Figure S3(a).

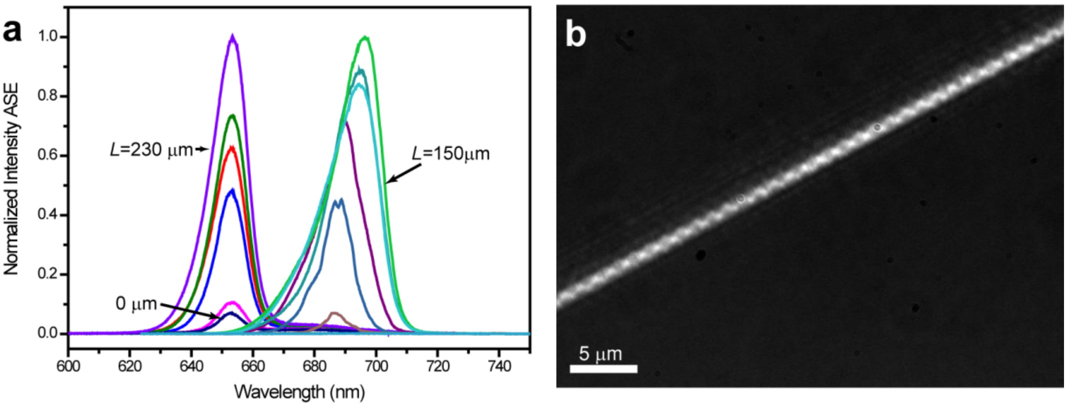

Figure S3. (a) The evolution of ASE intensities obtained from $CdSe_{0.625}S_{0.375}$ and CdSe nanorods under 20.45 mJ/cm^2 and 6.23 mJ/cm^2. The films were prepared by electric field producing aligned (b) Optical microscopy image of striped laser beam.

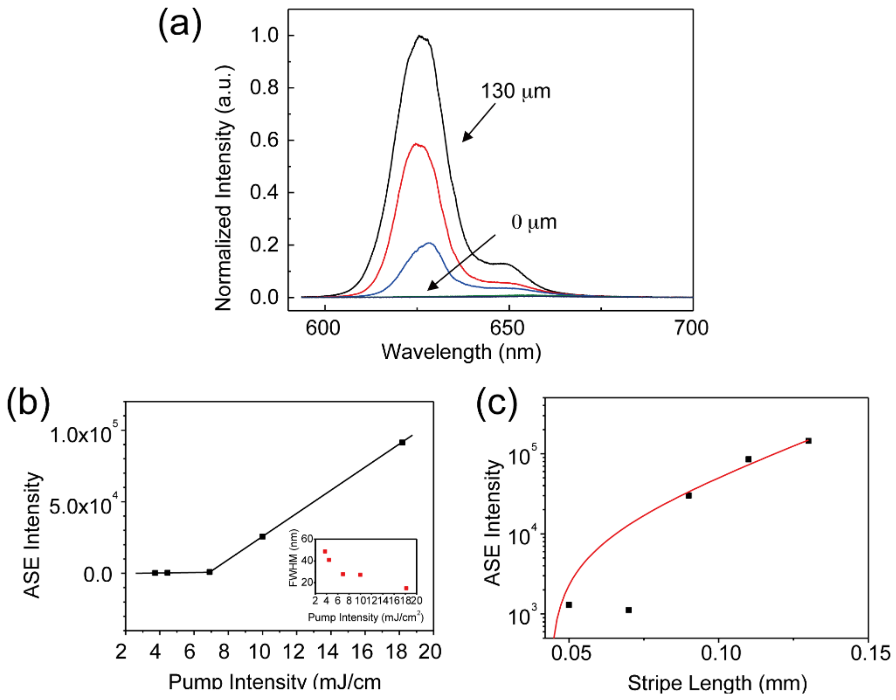

Figure S4. (a) ASE spectra obtained from $CdSe_{0.375}S_{0.625}$ (S-rich sample) excited over lengths between 0 and 130 um at 32.73 mJ/cm^2; (b) ASE intensities for $CdSe_{0.375}S_{0.625}$ nanorods at stripe length of 0.1 mm; the inset of (b): corresponded FWHM of data present in (b); (c) ASE intensities at pump intensity of 32.73 mJ/cm^2.

Comparison of ASE threshold of well aligned and disordered samples

To compare the ASE threshold of the well aligned and disordered samples, an optically smooth area (without cracks) is first identified by wide field optical image. Instead of stripe shaped laser beam, a Gaussian shaped laser spot is directly focused on the sample. The thickness of the ordered sample (Figure S5(a)) and disorder sample (Figure S5(b)) are both around 500 nm. PL spectra as a function of pump intensity are given in Figure S5(c). Compared to striped excitation, the ASE obtained this way required high pump intensity. Nevertheless, it clearly demonstrates that ASE threshold for the ordered sample occurs at lower value (590 mJ/cm^2) than 934 mJ/cm^2 of disordered sample.

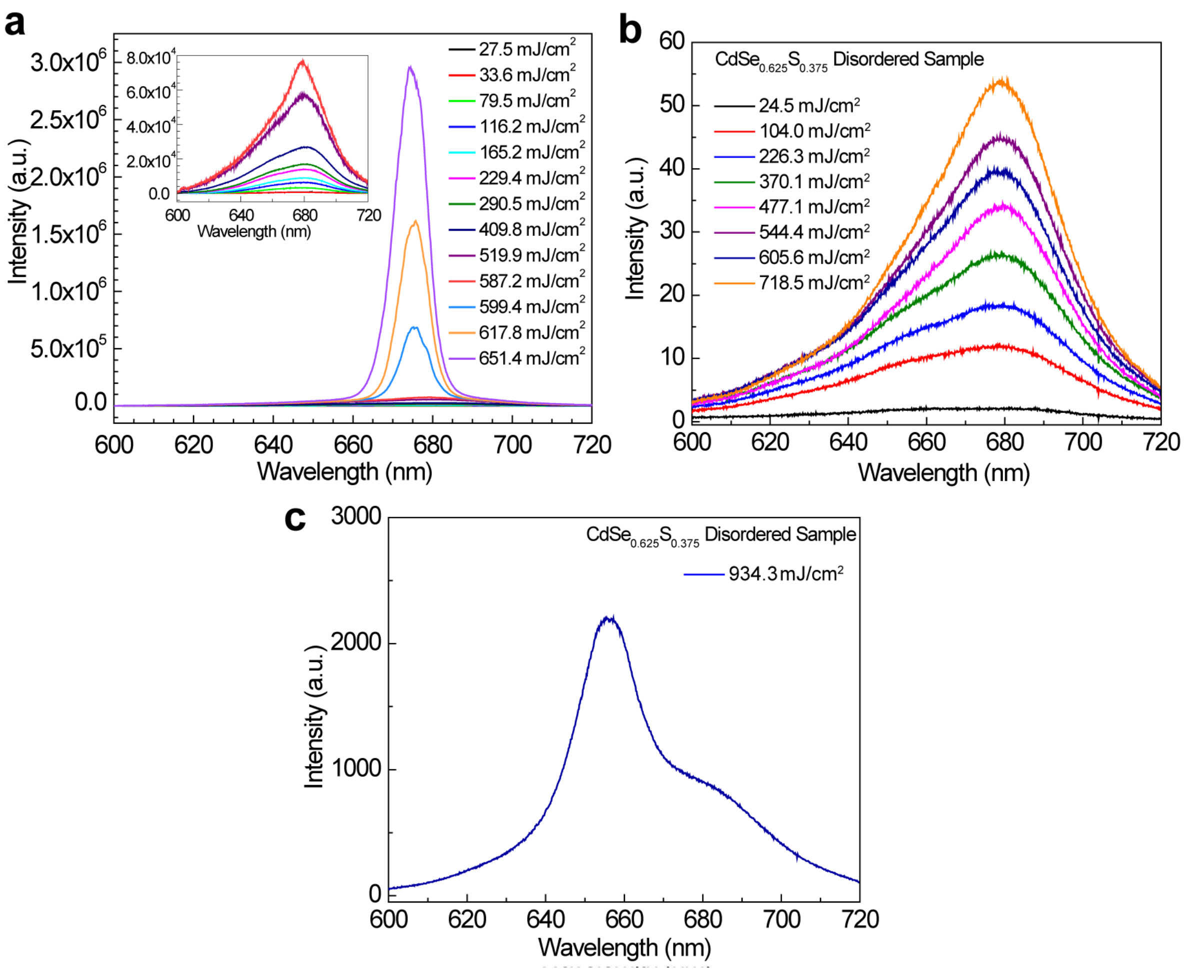

Figure S5. The evolution of PL spectrum of highly ordered (a) and disordered (b) $CdSe_{0.625}S_{0.375}$ film with excitation intensity up to 718.5 mJ/cm^2, when excited by a pulsed laser at 563 nm (1 KHz, ~ 4 ps pulse width). The ordered film showed apparent ASE spectra starting at 587.2 mJ/cm^2.(c) The onset of ASE observed on the disordered film at very high pump power.

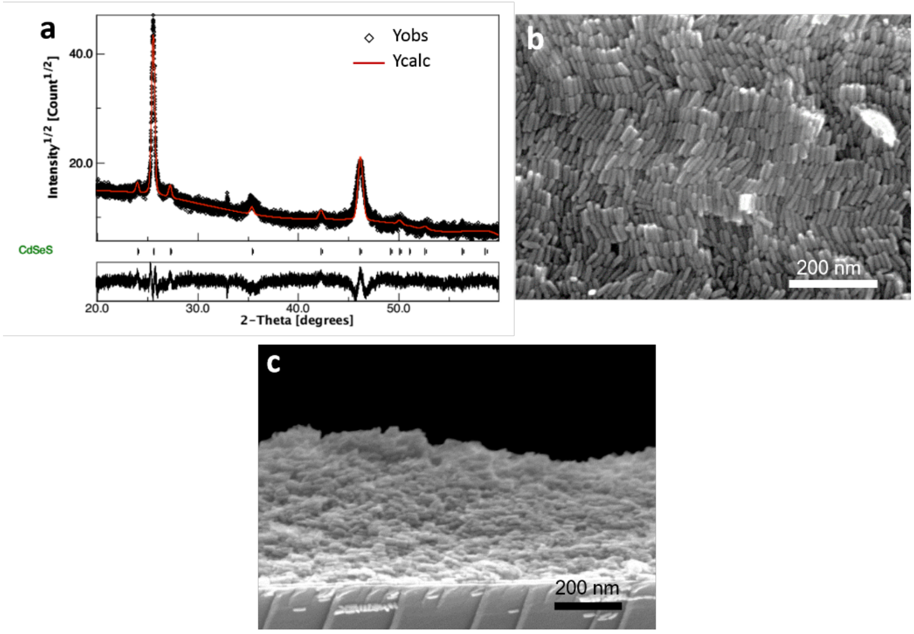

Figure S6. (a) Rietveld Refinement of the XRD pattern of the electric field aligned CdSeS (*x* = 0.625) nanorods. The refinement indicate that 83.7±6 % of the rods in the sample are parallel to the 002 direction. The SEM images of (b) ordered and (c) disordered samples.

Calculation of resonant modes in a squared cavity

Poon et al.[1] presented the calculation of resonant modes in a squared cavity, Ma et al.[2] used similar theoretical model to calculate the cavity modes in a plasmonic square cavity. The angle of internal reflection is shown in Figure S7.

$$(m_x + m_y)\lambda = 2na(\cos\theta + \sin\theta) \quad \text{(S1)}$$

Where the sum $(m_x + m_y)$ is the mode order, which are integer numbers, n is the refractive index and a is the length of the square. The low loss resonant cavity mode occurs when the total internal reflection boundary condition is satisfied. For the total internal reflection boundary, $\tan\theta_c < \tan\theta < \tan(90°-\theta_c)$.Given the 4-fold symmetry of the square, the number of modes in a given spectral range $\delta\nu$ is,

$$N \cong 2n_g n \frac{\delta\nu}{\nu}\left(\frac{L}{\lambda}\right)^2 \left[1 - \sin\left(\frac{\pi}{4} + \theta_c\right)\right] \quad \text{(S2)}$$

Where we have substituted for the cavity circumference, $L = 4a$. When evaluate this expression for the device shown in Figure 4(a) of the main text, with $\frac{\delta \nu}{\nu} = \frac{\delta \lambda}{\lambda} = 20/680$ (spectral range of CdSe emission); $n = 2.7$ (mode effective index,); $L = 40\ \mu m$; and $n_g = 6$, it is found that there are approximately $N \cong 500$ modes within the $CdSe_xS_{1-x}$ emission bandwidth when rounded to the nearest integer. This concurs with observation of 3 laser modes in the experiment and the predictions of the mode calculations in Figure 4. Note that this does not account for the possibility of modes to propagate either clockwise or anti-clockwise within the cavity.

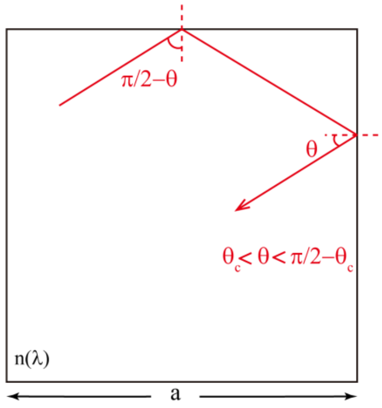

Figure S7. The rays trajectories of a four-bounce round-trip path in a square cavity with incident angles θ and 90°-θ at the orthogonal sidewall.

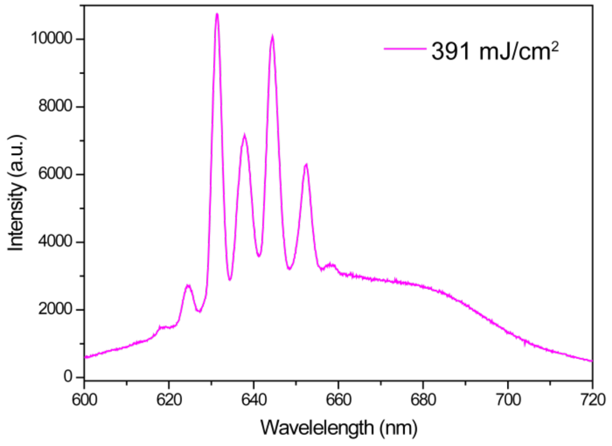

Figure S8. The evolution of PL spectra of another square cavity fabricated on orderd $CdSe_{0.625}S_{0.375}$ film shows the onset of lasing.

REFERENCES

1. Poon, A. W., Courvoisier, F. & Chang, R. K. Multimode resonances in square-shaped optical microcavities. *Opt. Lett.* **26,** 632 (2001).

2. Ma, R.-M., Oulton, R. F., Sorger, V. J., Bartal, G. & Zhang, X. Room-temperature sub-diffraction-limited plasmon laser by total internal reflection. *Nat. Mater.* **10,** 110–113 (2011).
